# Microstrip Triband Bandstop Fitler with Sharp Stop Band Skirts and Independently Controllable Second Stop Band Response

**DOI:** 10.1155/2014/760838

**Published:** 2014-06-12

**Authors:** Kishor Kumar Adhikari, Nam-Young Kim

**Affiliations:** RFIC Lab, Department of Electronics Engineering, Kwangwoon University, Nowon-Gu, Seoul 139-701, Republic of Korea

## Abstract

This paper presents a compact planar triband bandstop filter (TBBSF) with compact size and high selectivity. The structure of the filter incorporates two folded trisection stepped-impedance resonators (TSSIRs). One of these resonators is designed to operate at the first and third center frequencies and the other resonator is designed to operate at the second center frequency of the proposed filter. To achieve a compact size filter, alternating impedance lines of the resonators are folded widthwise and also one resonator is embedded within another. Theoretical analysis and design procedures are described, including the synthesis equations for each resonator. The main advantage of the proposed method is that the filter provides flexibility to tune the second center frequency and control the corresponding bandwidth without changing the first and third stop band response. Additionally, several reflection zeros (RZs) are introduced in the pass band to improve its flatness. To demonstrate the feasibility of the proposed design method, both the first and second order TBBSFs were designed, simulated, and fabricated, with center frequencies of 1.92 GHz, 3.55 GHz, and 5.5 GHz.

## 1. Introduction

There is an increasing demand for multiband bandstop filters (BSFs) in the rapidly growing modern multifunctional communication systems such as WiMax, Wireless Local Area Network (WLAN), and mobile communication system. The multiband BSFs used in the multiband transceivers of these systems effectively suppress the unwanted signals of different specific frequency bands. Compared to the single-band BSFs, these filters are more popular among the researchers because they can greatly reduce the circuit size and lower the cost of the product. Moreover, these filters have the additional advantages of low pass band insertion loss and minimal group delay [1].

Recently, researchers have been extensively investigating the use of triple-band bandstop filters (TBBSFs) [2–6] in the rapidly evolving WiMax and WLAN systems. The major design trends for these filters are compact size, high performance, and the flexibility to control the individual stop band response. Previous research has proposed several effective approaches for designing dual-band BSFs [7–11]. However, the design of a compact size TBBSF with good skirt selectivity and individually controlled stop bands still remains an ongoing challenge, which may be partially due to increased design complexities with more operation bands. Therefore, this paper mainly focuses on the design of a new-structure of a TBBSF with both compact size and sharp skirt selectivity. Additionally, the proposed work has paid more attention to add more flexibility to the control of the individual center frequency and its corresponding bandwidth, while also miniaturizing the size of the filter. The proposed structure of the TBBSF incorporates two parallel-connected trisection stepped-impedance resonators (TSSIRs) shunt-connected to a planar transmission line. One of these resonators creates the first and third stop band and the other inserts an individually controllable second stop band between them. To improve the selectivity of the filter, this paper also describes the second-order TBBSF, which is formed by replicating the similar combination of two TSSIRs with the same planar transmission line at a certain electrical separation from the first. The second-order filter exhibits both improved insertion losses and return losses, but at a price of an increase in the circuit size. Additionally, four open-end impedance lines, which are associated with the structure of the filter, include several reflection zeros (RZs) in the pass band to improve its flatness [12–14]. The designed second-order filter operates at 1.92 GHz, 3.55 GHz, and 5.50 GHz with corresponding bandwidths of 48.0%, 30.2%, and 19.1%, respectively, and can be a very good candidate for application in GSM-1900, WiMax, and WLAN systems.

## 2. Design and Theoretical Analysis

A TSSIR has alternating sections of very high and very low impedance lines. As a result, the resonator can support 90° simultaneously at multiple resonant frequencies and is very suitable for the realization of compact multiband BSFs [7–9]. However, if a single TSSIR is used to realize a TBBSF, three stop bands with a wide range of possible frequency gaps and possible bandwidths are very difficult to achieve because the change in electrical length or impedance of any section of such resonator causes change in more than one stop band response. Therefore, the use of two TSSIRs for realizing TBBSF is proposed in this work. One of these TSSIRs creates the first and the third stop bands with a wide gap and the other TSSIR is used to insert the tunable second stop band between them.

### 2.1. Configuration of the TSSIR and the TBBSF

**Figure 1:**
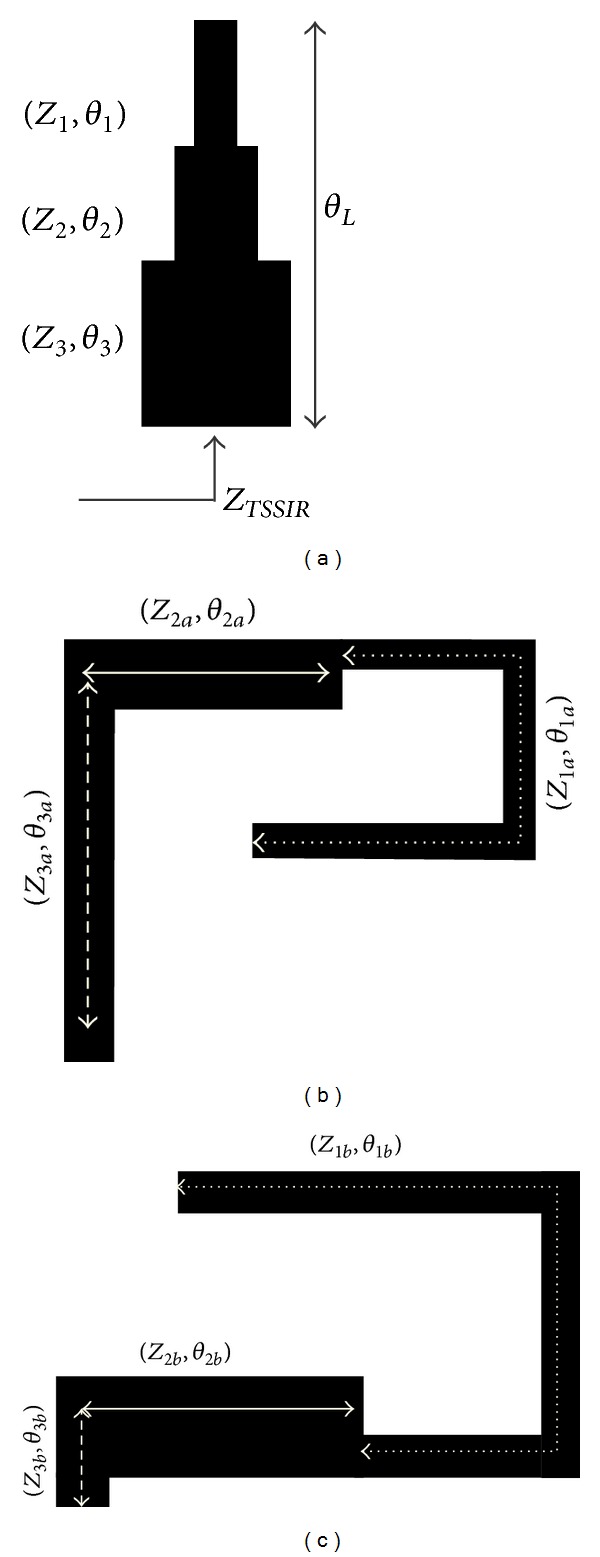
Configurations of (a) general trisection SIR, (b) proposed TSSIR_*a*_ resonating at the first and third center frequencies, and (c) proposed TSSIR_*b*_ resonating at the second center frequency.

**Figure 2:**
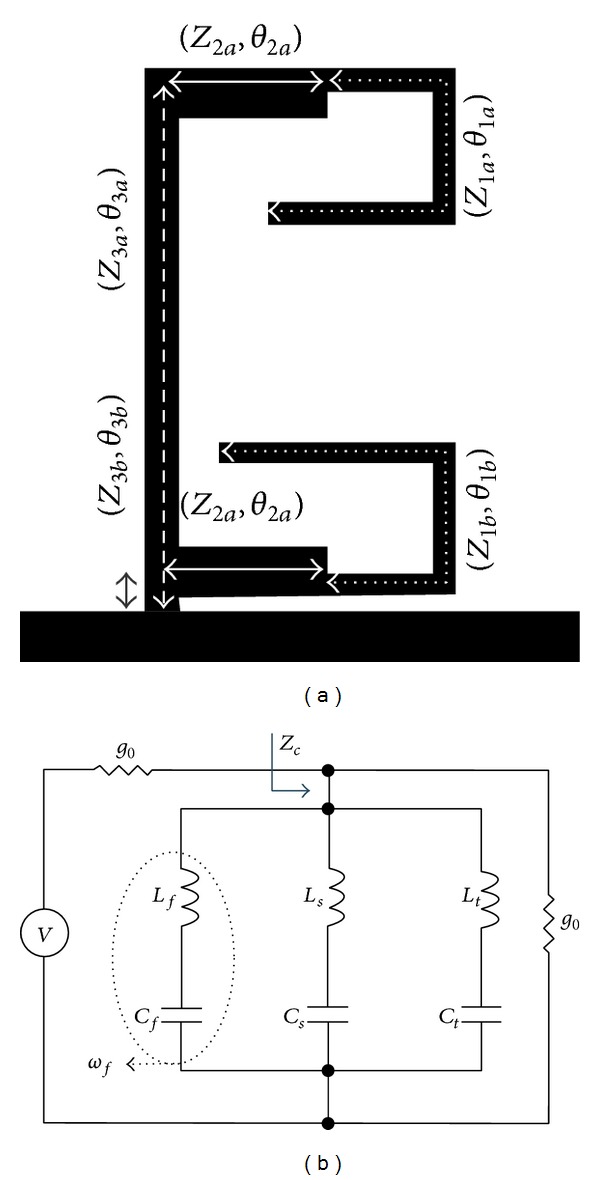
(a) Design layout of the proposed TBBSF of order, *N* = 1. (b) Equivalent circuit in terms of the shunt-connected series *LC* resonators.

**Figure 3:**
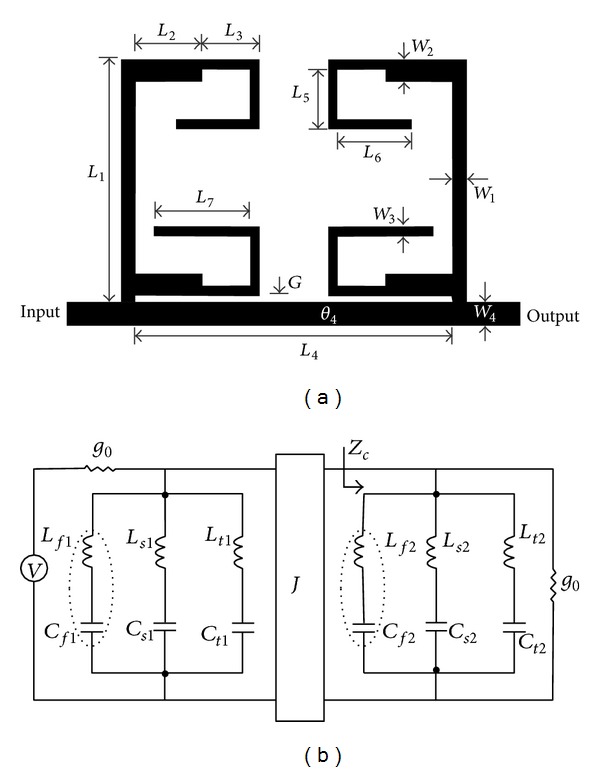
(a) Design layout of the proposed TBBSF of order, *N* = 2. (b) Equivalent circuit in terms of the shunt-connected series *LC* resonators and admittance (*J*) inverter.

The configuration of the general TSSIR is shown in Figure 1(a) and the proposed TSSIR_*a*_ with characteristic impedances and electrical lengths of (*Z*_1*a*_, *θ*_1*a*_), (*Z*_2*a*_, *θ*_2*a*_), and (*Z*_3*a*_, *θ*_3*a*_) are shown in Figure 1(b). TSSIR_*a*_ resonates at the first and third center frequencies of the proposed filter.  Figure 1(c) shows another proposed TSSIR_*b*_ with characteristic impedances and electrical lengths of (*Z*_1*b*_, *θ*_1*b*_), (*Z*_2*b*_, *θ*_2*b*_), and (*Z*_3*b*_, *θ*_3*b*_) and it resonates at the second center frequency of the proposed filter.  Figure 2(a) shows the proposed TBBSF with order *N* = 1. To realize this filter, TSSIR_*a*_ is first shunt-connected with a 50 Ω planar transmission line. TSSIR_*b*_ is then embedded within TSSIR_*a*_ in a way as shown in the figure that it does not increase the original circuit size of the filter.  Figure 2(b) models the equivalent circuit of Figure 2(a) using three series *LC* resonators connected in parallel. The resonators *L*_*f*_ *C*_*f*_, *L*_*s*_ *C*_*s*_, and *L*_*t*_ *C*_*t*_ resonate at the first, second, and third center frequencies denoted by *ω*_*f*_, *ω*_*s*_, and *ω*_*t*_, respectively. When the series resonator *L*_*f*_ *C*_*f*_ resonates at *ω*_*f*_, the input signal is shorted to ground, while series resonators *L*_*s*_ *C*_*s*_ and *L*_*t*_ *C*_*t*_ behave as an open circuit and have no influence on the input signal. A similar operation occurs when the resonators *L*_*s*_ *C*_*s*_ and *L*_*t*_ *C*_*t*_ resonate at *ω*_*s*_ and *ω*_*t*_, respectively, and thus the circuit acts as TBBSF. To improve the skirt selectivity of the filter, its order is increased to *N* = 2 by replicating the shunt connection of the similar combination of two TSSIRs at a separation of *θ*_4_ with the same transmission line, as shown in Figure 3(a).  Figure 3(b) models the equivalent circuit of Figure 3(a) using all-shunt-connected series *LC* resonators and an admittance inverter (*J* inverter).

Now, both of the *L*_*f*_ *C*_*f*_ resonators, shown by the dotted blocks in Figure 3(b), resonate at *ω*_*f*_, with the input signal shorted and opened, to form a stop band with a higher return loss. Similarly, the second and third stop bands with improved return losses are obtained using pair of series resonators *L*_*s*_ *C*_*s*_ and *L*_*t*_ *C*_*t*_, respectively. Based on the frequency mapping [15] from the low-pass prototype to the bandstop, the *LC* elements can be determined using *L*_*i*1_ = 1/*ω*_*i*_ *g*_1_Δ_*i*_, *C*_*i*1_ = *g*_1_Δ_*i*_/*ω*_*i*_ (for *LC* elements on the left side of the *J* inverter) and *L*_*i*2_ = *g*_2_Δ_*i*_/*ω*_*i*_, *C*_*i*2_ = 1/*ω*_*i*_ *g*_2_Δ_*i*_ (for *LC* elements on the right side of the *J* inverter). Here, the subscript *i* represents for the letters *f*, *s*, and *t*, which denote the first, second, and the third band, respectively, Δ_*f*_, Δ_*s*_, and Δ_*t*_ representing the corresponding bandwidths of the first, second, and third band, respectively, and *g*_1_( = 1.4142) and *g*_2_( = 1.4142) are the element values of the low-pass filter prototype. For the proposed TBBSF with center frequencies of 1.92 GHz, 3.55 GHz, and 5.5 GHz and corresponding fractional bandwidths of 60%, 35%, and 25%, respectively, the values of equivalent circuit components are *L*_*f*1_ = 4.88 nH, *L*_*s*1_ = 4.52 nH, *L*_*t*1_ = 4.09 nH, *C*_*f*1_ = 1.40 pF, *C*_*s*1_ = 0.44 pF, *C*_*t*1_ = 0.20 pF, *L*_*f*2_ = 3.51 nH, *L*_*s*2_ = 1.10 nH, *L*_*t*2_ = 0.51 nH, *C*_*f*2_ = 1.95 pF, *C*_*s*2_ = 1.81 pF, and *C*_*t*2_ = 1.63 pF.

The input impedance *Z*_TSSIR_ of a generalized trisection SIR can be expressed as

(1) ZTSSIR=jZ3(tanθ3−(Z2/Z3)((Z1/Z3)cot⁡θ1−(Z2/Z3)tan⁡θ2)((Z2/Z3)+(Z1/Z3)cot⁡θ1tan⁡θ2)) ×(1+(Z2/Z3)tan⁡θ3((Z1/Z3)cot⁡θ1−(Z2/Z3)tan⁡θ2)((Z2/Z3)+(Z1/Z3)cot⁡θ1tan⁡θ2))−1.

The impedance of the parallel-serial *LC* resonator *Z*_*c*_ in either Figures 2(b) or 3(b) can be derived as

(2) Zc=jωLfLsLt(ω2−ωf2)(ω2−ωs2)(ω2−ωt2)D,D=[ωfLsLtCf(ω2−ωs2)(ω2−ωt2) +ωsLfCsLt(ω2−ωt2)(ω2−ωf2) +ωtLsLfCt(ω2−ωf2)(ω2−ωs2)],

which does not take *g*_0_ into account.

### 2.2. Synthesis Equations and Analysis of Resonator Characteristics

The proposed TSSIR and its equivalent circuit should have the same reactance slope parameter, *x* = *ω*_*i*_/2 · *dX*/*dω*∣*ω*_*i*_ [16] to obtain the required bandwidths. Thus, the resonant condition *Z*_TSSIR_ = 0 and the reactance slope parameter at the two resonant frequencies can be used to obtain four simultaneous equations, which are given by

(3a) $$\begin{array}{c} \tan\theta_{3}=\frac{Z_{2}}{Z_{3}}\frac{\left(Z_{1}/Z_{3}\right)\text{cot}\;\theta_{1}-\left(Z_{2}/Z_{3}\right)\tan\theta_{2}}{\left(Z_{2}/Z_{3}\right)+\left(Z_{1}/Z_{3}\right)\text{cot}\;\theta_{1}\tan\theta_{2}}, \end{array}$$

(3b) tan⁡(rfθ3) =Z2Z3(Z1/Z3)cot⁡(rfθ1)−(Z2/Z3)tan⁡(rfθ2)(Z2/Z3)+(Z1/Z3)cot⁡(rfθ1)tan⁡(rfθ2),

(3c) (0.5Z3sec2θ3A) ×([(Z2Z3)+(Z1Z3)(tan⁡θ2+(Z2Z3)tan⁡θ3) ×cot⁡θ1−(Z2Z3)2tan⁡θ2tan⁡θ3]2)−1=1g1Δf,

(3d) (0.5Z3rfsec2(rfθ3)B) ×({(Z2Z3)+(Z1Z3)[tan⁡(rfθ2)+(Z2Z3)×tan⁡(rfθ3)]cot ⁡(rfθ1)−(Z2Z3)2tan⁡(rfθ2)tan⁡(rfθ3)}2)−1=1g1Δs,

(4) A=Z1Z3[(Z2Z3)2θ1+Z2Z3Z1Z3θ2+Z1Z3θ3]cot2 θ1tan2θ2+2Z2Z3Z1Z3[1−(Z2Z3)2]θ3cot ⁡θ1tan⁡θ2+Z2Z3Z1Z3×(Z2Z3θ1+Z1Z3θ2+Z2Z3Z1Z3θ3)cot2 θ1+(Z2Z3)2×[Z1Z3θ1+Z2Z3θ2+(Z2Z3)2θ3]tan2θ2+(Z2Z3)2(Z1Z3θ1+Z2Z3θ2+θ3),B=Z1Z3[(Z2Z3)2θ1+Z2Z3Z1Z3θ2+Z1Z3θ3]×cot2 (rfθ1)tan2(rfθ2)+2Z2Z3Z1Z3×[1−(Z2Z3)2]θ3cot⁡ (rfθ1)tan⁡(rfθ2)+Z2Z3Z1Z3(Z2Z3θ1+Z1Z3θ2+Z2Z3Z1Z3θ3)cot2 (rfθ1)+(Z2Z3)2[Z1Z3θ1+Z2Z3θ2+(Z2Z3)2θ3]tan2(rfθ2)+(Z2Z3)2(Z1Z3θ1+Z2Z3θ2+θ3),

where *r*_*f*_ is the frequency ratio *ω*_second_/*ω*_first_, *ω*_second_ is the second resonant frequency for TSSIR_*a*_ and third center frequency for the proposed TBBSF, and *θ*_1_, *θ*_2_, and *θ*_3_ are all specified at the *ω*_first_. To determine six design parameters (*Z*_1_, *Z*_2_, *Z*_3_, *θ*_1_, *θ*_2_, and *θ*_3_) of TSSIR using four simultaneous equations (3a), (3b), (3c), and (3d), *Z*_1_/*Z*_3_ and *Z*_2_/*Z*_3_ are taken as degrees of freedom. However, the resonant frequencies and associated bandwidths of the TSSIR realized with these parameters differ slightly from expected values, because they change with the variations in *Z*_1_/*Z*_3_ and *Z*_2_/*Z*_3_. Therefore, the design parameters obtained by solving (3a), (3b), (3c), and (3d) are optimized with the help of full wave EM simulator to get the expected results.

**Figure 4:**
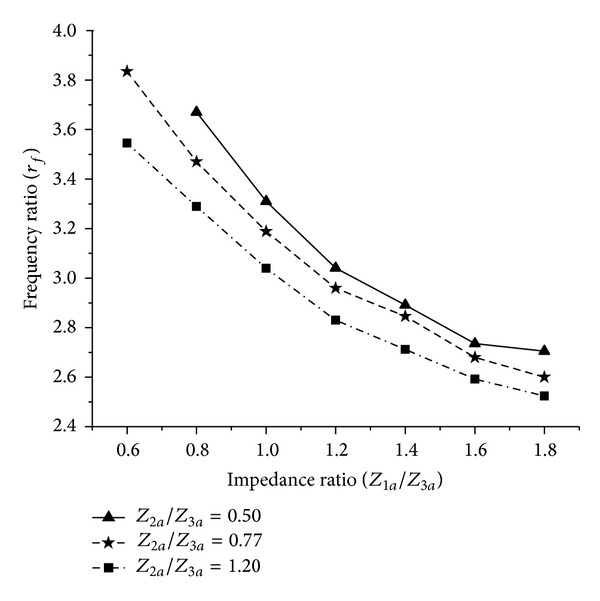
Variation of the frequency ratio (*r*_*f*_) versus varied impedance ratios for the proposed TSSIR_*a*_.

**Figure 5:**
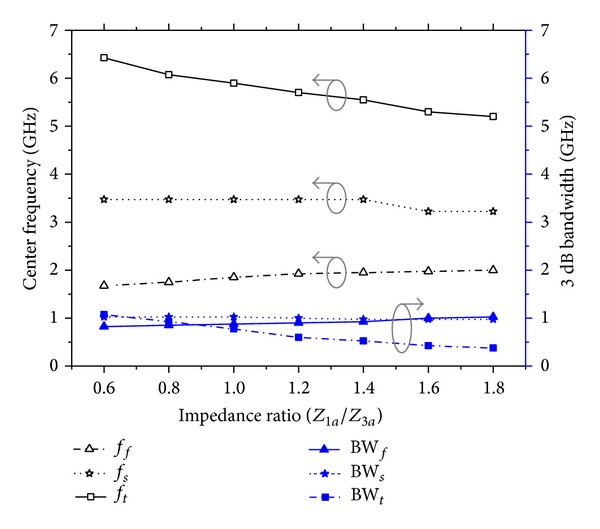
Plot of the variations of the center frequencies and bandwidths of the TBBSF versus the varied impedance ratio for the proposed TSSIR_*a*_ shows that *f*_*f*_, *f*_*t*_ and BW_*f*_, BW_*t*_ can be controlled at almost constant *f*_*s*_ and BW_*s*_, respectively.

In the proposed TBBSF, TSSIR_*a*_ is used to realize the first and the third stop bands. To achieve these stop bands, the design parameters *Z*_1*a*_, *Z*_2*a*_, *Z*_3*a*_, *θ*_1*a*_, *θ*_2*a*_, and *θ*_3*a*_ must be determined for the desired *ω*_*f*_ and *ω*_*t*_ and the corresponding bandwidths Δ_*f*_ and Δ_*t*_, respectively. However, only four simultaneous equations (3a), (3b), (3c), and (3d) are available. Therefore, the impedance ratios *Z*_1*a*_/*Z*_3*a*_ and *Z*_2*a*_/*Z*_3*a*_ are used as degrees of freedom. However, when *Z*_2*a*_/*Z*_3*a*_ = 1, TSSIR_*a*_ becomes a two-sectioned SIR. Therefore, *Z*_1*a*_/*Z*_3*a*_ is varied from 0.6 to 1.8 (taking the structure of the proposed filter and the practical value of *Z*_high_ = 125 Ω and *Z*_low_ = 20 Ω into account) for *Z*_2*a*_/*Z*_3*a*_ = 0.50 (<1), 0.77 (<1), and 1.2 (>1) for the parametric study of the design parameters of the TSSIR_*a*_. The remaining four parameters *Z*_3*a*_, *θ*_1*a*_, *θ*_2*a*_, and *θ*_3*a*_ can be obtained by solving the available four simultaneous equations by using a root-searching program. Additionally, the resonator behavior is much guided by open-end impedance line and, therefore, the aforementioned range of *Z*_1*a*_/*Z*_3*a*_ is obtained by varying *Z*_1*a*_ at a fixed *Z*_3*a*_. The open-end line of TSSIR acts as equivalent inductor or capacitor depending upon the value of *θ*_1*a*_ relative to *θ*_3*a*_. If *θ*_3*a*_ > *θ*_1*a*_, the change in *Z*_1*a*_ causes more effective changes in *L*_*f*_ and *L*_*t*_ compared to *C*_*f*_ and *C*_*t*_. Therefore, the changes in only *L*_*f*_ and *L*_*t*_ are considered to describe the change in the first and third stop band response of the TBBSF. However, the change in *Z*_1*a*_ causes more effective changes in *C*_*f*_ and *C*_*t*_ compared to *L*_*f*_ and *L*_*t*_, provided that *θ*_3*a*_ < *θ*_1*a*_. Therefore, the changes in only *C*_*f*_ and *C*_*t*_ are considered to describe the change in stop band response of the proposed filter for this case. The variation of *r*_*f*_ for the varied impedance ratios is shown in Figure 4, which indicates that *r*_*f*_ can be varied within a maximum range from 2.60 to 3.84 when *Z*_2*a*_/*Z*_3*a*_ = 0.77. To carry out the further analysis of the proposed TSSIR_*a*_, *Z*_2*a*_/*Z*_3*a*_ is, therefore, maintained at 0.77. The proposed structure of the filter requires higher value of *θ*_3*a*_ for allowing TSSIR_*b*_ to be embedded within TSSIR_*a*_, which must be maintained while determining the design parameters for TSSIR_*a*_. However, higher value of *θ*_3*a*_ causes TSSIR_*a*_ to be more inductive than capacitive, and, therefore, changes in *Z*_1*a*_/*Z*_3*a*_ result in more effective changes in *L*_*f*_ and *L*_*t*_ (from the equivalent circuit in Figure 2(a)) compared to *C*_*f*_ and *C*_*t*_.  Figure 5 plots the center frequencies of the filter for the variations in *Z*_1*a*_/*Z*_3*a*_ and shows that *f*_*f*_ and *f*_*t*_ increase and decrease, respectively, with increase in *Z*_1*a*_/*Z*_3*a*_. Thus, *r*_*f*_ decreases with an increase in *Z*_1*a*_/*Z*_3*a*_. This result is explained more clearly with the help of equivalent shunt-connected series *LC* circuit shown in Figure 2(b). For a shunt-connected series *LC* circuit, resonance frequency and corresponding 3 dB bandwidth can be, respectively, expressed as

(5) $$\begin{array}{c} f_{0}=\frac{1}{2\pi {\left(LC\right)}^{1/2}}, \end{array}$$

(6) $$\begin{array}{c} \Delta f_{3\;\text{dB}}=\frac{Z_{0}}{2\pi L}=4\pi^{2}f_{0}^{2}Z_{0}C. \end{array}$$

Therefore, the increase and decrease in *f*_*f*_ and *f*_*t*_ are expected according to (5) because of respective decrease and increase in *L*_*f*_ and *L*_*t*_ with an increase in *Z*_1*a*_/*Z*_3*a*_.  Figure 5 also plots the changes in the bandwidths for different stop bands with changes in *Z*_1*a*_/*Z*_3*a*_. The BW_*f*_ and BW_*t*_ increase and decrease according to (6), respectively, because increase in *Z*_1*a*_/*Z*_3*a*_ causes respective decrease and increase in *L*_*f*_ and *L*_*t*_. However, BW_*s*_ remains almost unchanged.

**Figure 6:**
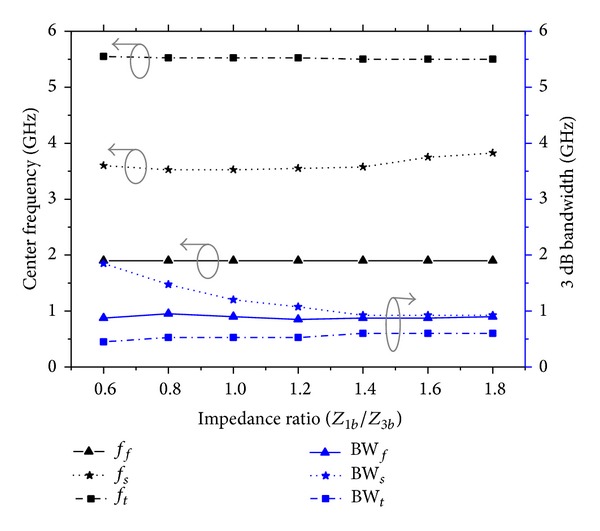
Plot of the variation of the center frequencies and bandwidths of the TBBSF versus the varied impedance ratio for the proposed TSSIR_*b*_ shows that *f*_*s*_ and BW_*f*_ can be controlled at almost constant *f*_*f*_, *f*_*t*_ and BW_*f*_, BW_*t*_, respectively.

In the proposed TBBSF, TSSIR_*b*_, which introduces the second stop band in the response of the filter, is shunted to the *Z*_3*a*_ of TSSIR_*a*_ to share the same impedance section. If a uniform impedance resonator (UIR) is used to replace TSSIR_*b*_ to simplify the design and analysis of the TBBSF, the necessary condition of *Z*_3*b*_ = *Z*_3*a*_ always results in single value of *Z*_3*b*_. Even if *Z*_2*b*_ = *Z*_1*b*_ ≠ *Z*_3*b*_ is used, it becomes two-section SIR. However, UIR with a fixed impedance value cannot be used to control the bandwidth of second stop band effectively. Therefore, the use of TSSIR_*b*_ is preferred to realize the second stop band instead of UIR. Additionally, TSSIR_*b*_ can also be used as UIR with all impedance sections having equal characteristic impedance. For analyzing TSSIR_*b*_, *Z*_1*b*_/*Z*_3*b*_ and *Z*_2*b*_/*Z*_3*b*_ are used as degrees of freedom. *Z*_1*b*_/*Z*_3*b*_ is varied from 0.6 to 1.8 for *Z*_2*b*_/*Z*_3*b*_ = 0.77 for a predetermined *Z*_3*b*_ and a fixed *θ*_3*b*_. The remaining two parameters *θ*_1*b*_ and *θ*_2*b*_ can be obtained by solving (3a) and (3c). The proposed structure of the filter causes lower value of *θ*_3*b*_, which must be maintained while determining the design parameters for TSSIR_*b*_. As a result, *θ*_3*b*_ can be shorter than *θ*_1*b*_ for the proposed TSSIR_*b*_.  Figure 6 plots the center frequencies of the TBBSF with the variation in *Z*_1*b*_/*Z*_3*b*_, which indicates that *f*_*s*_ increases with an increase in *Z*_1*b*_/*Z*_3*b*_. This behavior is expected, because *C*_*s*_ (from the equivalent circuit) increases with an increase in *Z*_1*b*_/*Z*_3*b*_. However, *f*_*f*_ and *f*_*t*_ remain unaffected with an increase in *Z*_1*b*_/*Z*_3*b*_.  Figure 6, which also plots the variations in the bandwidth for different stop bands with the changes in *Z*_1*b*_/*Z*_3*b*_, indicates that BW_*f*_ and BW_*t*_ remain almost the same, while BW_*s*_ decreases rapidly with an increase in *Z*_1*b*_/*Z*_3*b*_.

**Figure 7:**
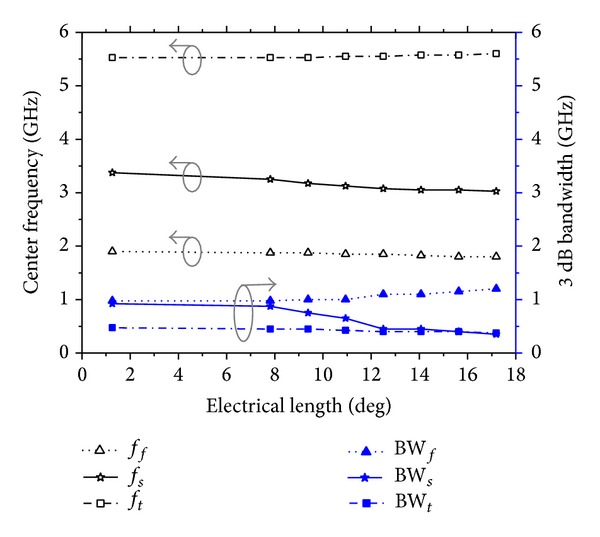
Plot of the variation of the center frequencies and bandwidths of the TBBSF versus the varied electrical length (*θ*_3*b*_) for the proposed TSSIR_*b*_ shows that *f*_*s*_ and BW_*f*_ can be controlled at almost constant *f*_*f*_, *f*_*t*_ and BW_*f*_, BW_*t*_, respectively.

To control the second stop band response of the proposed TBBSF, *θ*_3*b*_ can be varied from 1.26° to 17.19° (calculated at *ω*_*f*_) for *Z*_1*b*_/*Z*_3*b*_ = 1.23 and *Z*_2*b*_/*Z*_3*b*_ = 0.77. This capability is one of the main advantages of the proposed filter structure, because *θ*_3*b*_ can be varied in the prestated range of values without changing the structure and size of the filter. When *θ*_3*b*_ increases, the total electrical length of TSSIR_*b*_ also increases, which results in an increase in *L*_*s*_ of the equivalent series *L*_*s*_ *C*_*s*_ circuit. Consequently, *f*_*s*_ and BW_*s*_ plotted in Figure 7 decrease according to (5) and (6), respectively. However, *f*_*f*_, *f*_*t*_, BW_*f*_, and BW_*t*_ remain almost unchanged.

### 2.3. Triband Bandstop Filter Design

**Figure 8:**
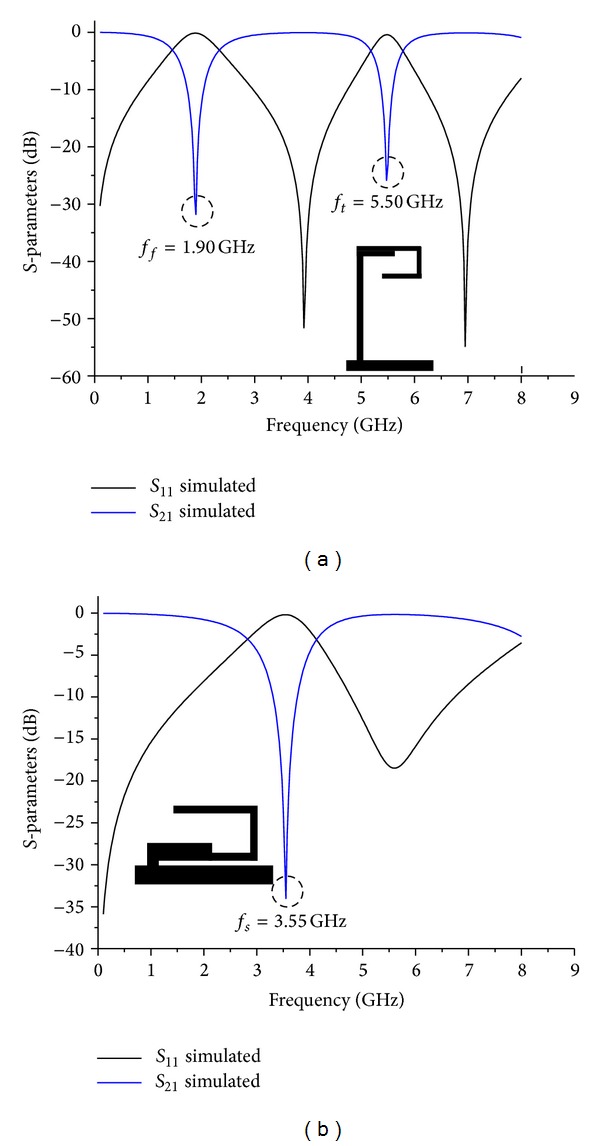
Simulated results of the proposed TSSIR_*a*_ and TSSIR_*b*_ shunt-connected to 50 Ω planar transmission line: (a) TSSIR_*a*_ (b) TSSIR_*b*_.

**Figure 9:**
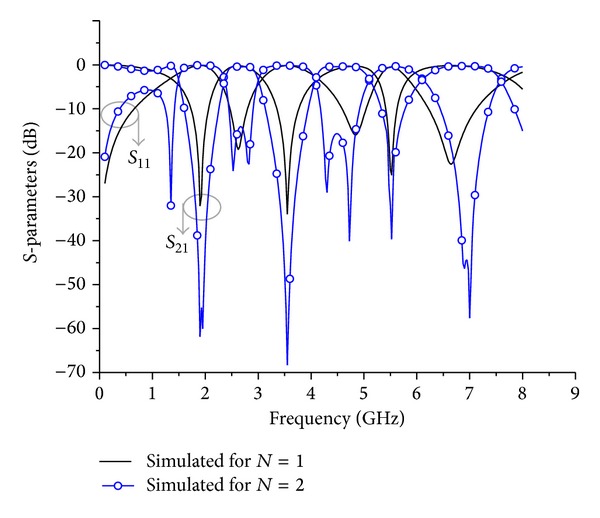
Simulated results of the proposed TBBSFs for different orders of *N* = 1 and *N* = 2.

By using the proposed design method, a filter was designed to operate at the center frequencies of 1.92 GHz, 3.55 GHz, and 5.5 GHz with the corresponding bandwidths of Δ_*f*_ = 60%, Δ_*s*_ = 35%, and Δ_*t*_ = 25%, respectively. For this, TSSIR_*a*_ shunt-connected to a 50 Ω planar transmission line with optimized design parameters *Z*_1*a*_ = 85.57 Ω, *Z*_2*a*_ = 53.34 Ω, *Z*_3*a*_ = 69.4 Ω, *θ*_1*a*_ = 34.2°, *θ*_2*a*_ = 13.15°, and *θ*_3*a*_ = 46.47° and TSSIR_*b*_ shunt-connected to a 50 Ω planar transmission line with optimized design parameters *Z*_1*b*_ = 85.57 Ω, *Z*_2*b*_ = 53.34 Ω, *Z*_3*b*_ = 69.4 Ω, *θ*_1*b*_ = 38.6°, *θ*_2*b*_ = 13.15°, and *θ*_3*b*_ = 1.26° were simulated separately and the simulated results are illustrated in Figure 8. The design parameters for both TSSIRs were evaluated at 1.92 GHz. The designed TSSIR_*a*_ and TSSIR_*b*_ were combined together to obtain a TBBSF of *N* = 1 and *N* = 2 as shown in Figures 2(a) and 3(a), respectively. The electrical length of the simplified admittance inverter between the TSSIRs for the second order filter can be calculated using *θ*_4_ = *nπ*/(*r*_*f*_ + 1) [17], where *n* = 1,2, 3…, yielding *θ*_4_ = 63.1°. The bandwidths for different stop bands of TBBSF depend much on *θ*_4_ and, therefore, it was optimized by the simulator to a value of 62.28°. The detailed physical dimensions of the TBBSF of order *N* = 2 illustrated in Figure 3(a) are *L*_1_ = 14.48 mm, *L*_2_ = 4 mm, *L*_3_ = 3.42 mm, *L*_4_ = 20.68 mm, *L*_5_ = 3.55 mm, *L*_6_ = 4.42 mm, *L*_7_ = 5.74 mm, *W*_1_ = 0.84 mm, *W*_2_ = 1.3 mm, *W*_3_ = 0.56 mm, *W*_4_ = 1.4 mm, and *G* = 0.4 mm. The calculated physical length of *θ*_1*a*_ = 34.2° is 10.544 mm. However, the physical dimensions in Figure 3(a) depict that *L*_3_ + *L*_5_ + *L*_6_ = 11.39 mm. The same situation exists for *θ*_1*b*_ = 38.6°. The calculated physical length is 11.90 mm. However, the physical dimensions result in *L*_3_ + *L*_5_ + *L*_7_ = 12.71 mm. The approximate difference of 0.8 mm appears, because the electrical lengths are calculated neglecting the bending losses at two bends of 0.56 mm wide open-end lines of TSSIR_*a*_ and TSSIR_*b*_. The designed filters were simulated using a full-wave EM simulator, Sonnet, and the simulated results of both filters are shown in Figure 9. The observation of simulation results depicts that the first order TBBSF has center frequencies of 1.9 GHz, 3.55 GHz, and 5.5 GHz with the corresponding bandwidths of Δ_*f*_ = 48%, Δ_*s*_ = 29%, and Δ_*t*_ = 10%, respectively. The second order TBBSF has center frequencies of 1.92 GHz, 3.55 GHz, and 5.5 GHz with the corresponding bandwidths of Δ_*f*_ = 48%, Δ_*s*_ = 30%, and Δ_*t*_ = 19%, respectively. The comparison of the simulated results for both TBBSFs indicates that the second order filter exhibits noticeably improved performance with higher return losses. Additionally, this filter has sharper transition caused by seven reflection zeros introduced in the pass band at 1.35 GHz, 2.52 GHz, 2.82 GHz, 4.30 GHz, 4.73 GHz, 6.90 GHz, and 7 GHz by the increased number of symmetric open-end stubs.

## 3. Implementation and Measurement 

**Figure 10:**
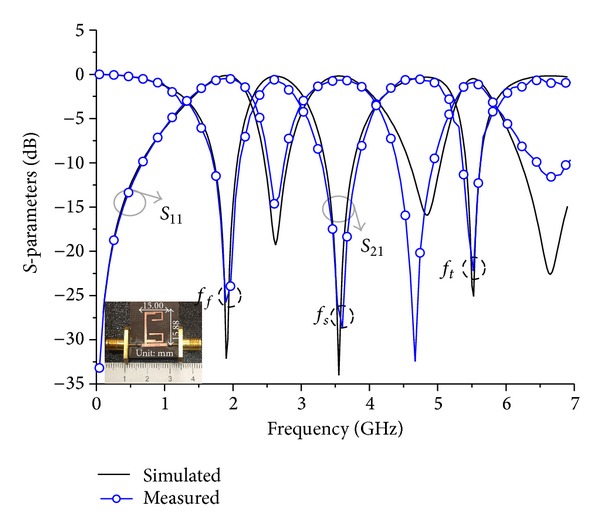
Photograph of the fabricated TBBSF (15 mm × 15.88 mm) and results of order *N* = 1.

**Figure 11:**
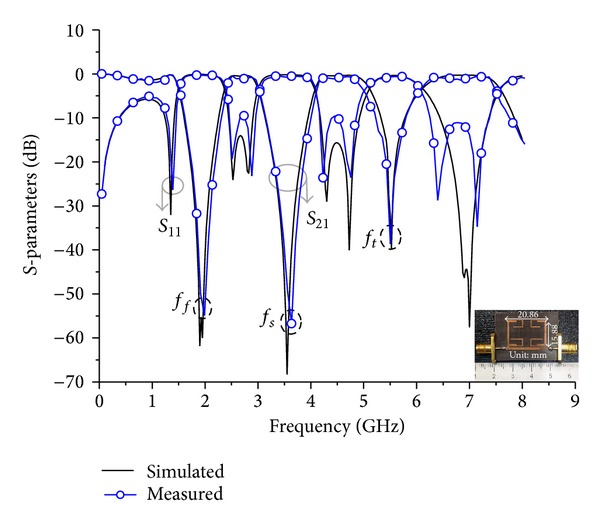
Photograph of the fabricated TBBSF (20.68 mm × 15.88 mm) and results of order *N* = 2.

To validate the proposed design concept, the designed TBBSFs of both the first and second order were fabricated on a Teflon substrate with dielectric constant (*ε*_*r*_) = 2.52, thickness (*h*) = 0.504 mm, and a loss tangent of 0.02. The fabricated filters were then measured using an Agilent 8510C VNA instrument. Figures 10 and 11 show the simulated and measured responses of the TBBSF of first and second order, respectively, which indicate that both responses have good agreement between the simulation results and the measurements. The center frequencies of the first order filter were measured to be *f*_*f*_ = 1.93 GHz, *f*_*s*_ = 3.61 GHz, and *f*_*t*_ = 5.50 GHz, with respective fractional bandwidths of 50.7%, 27.9%, and 11.2%. The center frequencies of the second order filter were measured to be *f*_*f*_ = 1.93 GHz, *f*_*s*_ = 3.61 GHz, and *f*_*t*_ = 5.50 GHz, with respective fractional bandwidths 46.46%, 28.1%, and 18.62%. The slight deviation observed in the measured results compared to simulated results was attributed to the dielectric substrate loss and the unexpected tolerances in the fabrication and soldering of the ports, which were not modeled during the simulation of the proposed filter [18, 19]. Additionally, an admittance (*J*) inverter is an ideal inverter with an electrical length of 90° at all frequencies. However, a homogeneous transmission line of fixed length (*θ*_4_) cannot act as ideal *J* inverter at all three stop bands [20]. So frequency shift and bandwidth variation in some stop bands occurred.

The observation of Table 1, which summarizes the simulation and measurement results of the TBBSFs and the comparison of the proposed filters with the other literature in Table 2, indicates that we have obtained a compact size filter with very good insertion and return losses using a similar value of the dielectric constant and relatively lower value of substrate thickness.

## 4. Conclusions

This paper described TBBSFs based on folded TSSIRs, whose characteristics are well investigated in terms of varying of the impedance ratios and changing the electrical length with the help of both mathematical relationships and simulation results for designing TBBSFs. The center frequencies and corresponding BWs of the proposed filter were shown to be conveniently varied over a wide range without changing the effective size of the filter. Additionally, the second order filter exhibits improved frequency selectivity with minimum return loss of 38 dB for each stop band and improved flat pass band response, but it has a drawback of an increased circuit size. The designed second order filter features three stop bands at 1.92 GHz, 3.55 GHz, and 5.50 GHz with corresponding fractional bandwidths of 48%, 30.2%, and 19.1%, respectively, which is suitable for GSM, WiMAX, and WLAN applications.

## Figures and Tables

**Table 1:** Performance comparison of simulated and measured results of the proposed TBBSF.

Parameter	Simulation	Measurement
TBBSF of order *N* = 1		
Center frequencies (GHz)	1.90, 3.55, 5.50	1.93, 3.61, 5.50
Insertion loss (IL) (dB)	−0.20, −0.28, −0.55	−0.50, −0.67, −0.93
Return loss (RL) (dB)	−32.4, −34.1, −25.1	−25.8, −28.7, −22.3
BW at −3 dB	0.92, 1.03, 0.55	0.98, 1.01, 0.62

TBBSF of order *N* = 2		
Center frequencies (GHz)	1.92, 3.55, 5.50	1.98, 3.63, 5.50
IL (dB)	−0.15, −0.32, −0.32	−0.34, −0.55, −0.68
RL (dB)	−55.4, −68.4, −39.8	−54.8, −56.7, −38.6
BW at −3 dB	0.92, 1.08, 1.02	0.92, 1.02, 1.08

**Table 2:** Performance comparison of reported TBBSFs.

Reference	Resonant frequency (GHz)	IL (dB)/RL (dB)	Substrate *ϵ*_*r*_, *h* (mm)	Size (*λ*_*g*_ × *λ*_*g*_)
This work (*N* = 1)	**1.93, 3.61, 5.50**	**−0.50, −0.67, −0.93/−25.8, −28.7, −22.3**	**2.52, 0.504**	**0.14 × 0.15**
This work (*N* = 2)	**1.98, 3.63, 5.50**	**−0.34, −0.55, −0.68/−54.8,−56.7, −38.6**	**2.52, 0.504**	**0.19 × 0.15**
Literature [2]	2.37, 3.54, 5.01	−0.35, −0.50, −0.92/−31.2, −30.9, −40.5	2.55, 1.5	0.44 × 0.18
Literature [3]	2.40, 3.50, 5.20	−1.5, −2.54, −1.16/−15, −13, −20	2.2, 1.575	0.21 × 0.11
Literature [4]	2.59, 6.88, 10.67	−0.40, −0.90, −1.10/−29.9, −28.2, −26.6	2.52, 0.504	0.21 × 0.11
